# Prevalence and factors associated with triple burden of malnutrition among mother-child pairs in India: a study based on National Family Health Survey 2015–16

**DOI:** 10.1186/s12889-021-10411-w

**Published:** 2021-02-23

**Authors:** Pradeep Kumar, Shekhar Chauhan, Ratna Patel, Shobhit Srivastava, Dhananjay W. Bansod

**Affiliations:** International Institute for Population Sciences, Mumbai, Maharashtra 400088 India

**Keywords:** Triple burden, Malnutrition, Mother-child pairs, NFHS, India

## Abstract

**Background:**

Malnutrition in mothers as well as in children is a significant public health challenge in most of the developing countries. The triple burden of malnutrition is a relatively new issue on the horizon of health debate and is less explored among scholars widely. The present study examines the prevalence of the triple burden of malnutrition (TBM) and explored various factors associated with the TBM among mother-child pairs in India.

**Methods:**

Data used in this study were drawn from the fourth round of the National Family Health Survey (NFHS-IV) conducted in 2015–16 (*N* = 168,784). Bivariate and binary logistic regression analysis was used to quantify the results. About 5.7% of mother-child pairs were suffering from TBM.

**Results:**

Age of mother, educational status of the mother, cesarean section delivery, birth size of baby, wealth status of a household, and place of residence were the most important correlates for the triple burden of malnutrition among mother-child pairs in India. Further, it was noted that mothers with secondary education level (AOR: 1.15, CI 1.08–1.23) were having a higher probability of suffering from TBM, and interestingly the probability shattered down for mothers having a higher educational level (AOR: 0.90, CI 0.84–0.95). Additionally, mother-child pairs from rich wealth status (AOR: 1.93, CI 1.8–2.07) had a higher probability of suffering from TBM.

**Conclusion:**

From the policy perspective, it is important to promote public health programs to create awareness about the harmful effects of sedentary lifestyles. At the same time, this study recommends an effective implementation of nutrition programs targeting undernutrition and anemia among children and obesity among women.

## Background

The prevalence of malnutrition is declining around the world. Still, the world is home to around 155 million stunted children and 52 million wasted children [1]. Malnutrition is a major public health concern for the developing world, specifically India [2]. India continues to be the largest contributor to the global prevalence of malnutrition. Malnutrition can critically affect child growth and development, along with child survival. Malnutrition is one of the established causes of morbidity and mortality among children worldwide [3]. In technical terms, malnutrition is understood as “bad nutrition,” and it includes both over and undernutrition [4]. Countries are struggling with malnutrition on the one hand, and on the other hand, obesity is becoming a concern [5]. Since long obesity was considered a concern in a developed country, recently developing countries like India are also observing the high prevalence of obesity among children [5, 6] and mothers [7] due to rapid food habits and lifestyle changes. Previous studies have focused on the coexistence of obesity and undernutrition, i.e., the double burden of malnutrition. Researchers have explored various forms of the double burden of malnutrition; underweight and obesity among mothers [8, 9], obesity and thinness among children [10, 11], underweight and obesity among children [12, 13], underweight and obesity among mothers as well as children [7].

Recently, the double burden of malnutrition was accompanied by micronutrient deficiency [14]. Micronutrient deficiency is studied extensively in relation to anemia among children in India [15]. Anemia among children has been a significant health concern for a long and is one of the significant nutrition-related morbidities in developing countries [16]. It is estimated that 58% of the children aged 6–59 months were anemic in India [15]. As far as developing countries are concerned, India contribute significantly to child anemia [17, 18]. Few studies have highlighted the coexistence of the triple burden of malnutrition (TBM) in children, i.e., undernutrition, micronutrient deficiency, and obesity [19]. TBM has also co-existed at the household level where the mother was obese, and her children were found to be either anemic or undernourished; undernourishment was measured with stunting, underweight, and wasting [20].

Malnutrition in mothers as well as in children is a significant public health challenge in most of the developing countries. The triple burden of malnutrition is a relatively new issue on the horizon of healthy debate and is less explored among scholars widely. The double burden of malnutrition has been studied significantly in the developing world [21, 22], including India [7, 23], but the domain of triple burden is yet to be explored fully. Despite a rising coexistence of various forms of maternal and child malnutrition [1], studies related to the TBM are negligible. Even in the same household, the coexistence of undernutrition and obesity is often seen as paradoxical, but there are quite a few explanations for this paradox. As food resources become meagre, people tend to eat low-cost, unhealthy, and highly energy-dense foods, such choices lead to household members becoming overweight and undernourished at the same time [1]. Most countries experience multiple burdens of malnutrition, that are being expressed as a combination of undernutrition among children and obesity among adults [1]. Therefore, child undernutrition and adult obesity (overweight/obese mothers) form the pair to depict TBM in this paper. This study explores the coexistence of TBM among mother-child pairs residing in the same household. For this study, anemic and undernourished (either stunted or wasted or underweight child) children, along with obese mothers, formed the mother-child pairs for the TBM in a household. In previous studies, the triple burden was measured with a combination of anemic and undernourished children and obese women [20]. This study’s primary objective is to examine the prevalence of TBM and explore various factors associated with TBM in India.

## Methods

This study’s data were drawn from the fourth round of the National Family Health Survey (NFHS) conducted in 2015–16, a cross-sectional national representative survey, to estimate the TBM and its associated factors among mother-child pairs. The NFHS 2015–16, conducted under the stewardship of the Ministry of Health and Family Welfare (MoHFW) of India, provides detailed information on population, health, and nutrition, for India as a whole, as well as for each state (29) and union territory (7) and district (640). For selecting Primary Sampling Units (PSUs), the 2011 census served as the sampling frame. A more detailed methodology of the NFHS-4 has been published in the report [24]. This study used anthropometric indices such as height-for-age, weight-for-height, and weight-for-age and hemoglobin levels to evaluate children’s nutritional status below 5 years of age (0–59 months). Children suffering from stunting, wasting, and underweight was defined as children with Z-scores below − 2 standard deviation for height-for-age (HAZ), weight-for-height (WHZ), and weight-for-age (WAZ), respectively [20]. For this study, blood hemoglobin level was categorized as anemic (< 11 g/dl) and not anemic (> = 11 g/dl). In addition, the study used body mass index (BMI) of women and according to WHO cut-off values (underweight: < 18.5 kg/ m^2;^ normal BMI: 18.5 to < 24.99 kg/m^2^ and overweight/obesity: > = 25.0 kg/m^2^) [24].

### Outcome variables

First, this study dichotomized all the dependent variables into two categories: the presence of malnutrition was coded as ‘1’, and the absence of malnutrition was coded as ‘0’. The study made four different combinations of malnutrition, which were: overweight/obese mother and wasted child (OM/WC), overweight/obese mother and stunted child (OM/SC), overweight/obese mother and underweight child (OM/UC), and overweight/obese mother and anemic child (OM/AC) in the same household. Further, these four categories were combined and grouped into a single category: overweight/obese mother and undernourished child (stunting/wasting/underweight) who was also anemic, which was considered as the triple burden of malnutrition (TBM) [20].

### Exposure variables

**Fig. 1:**
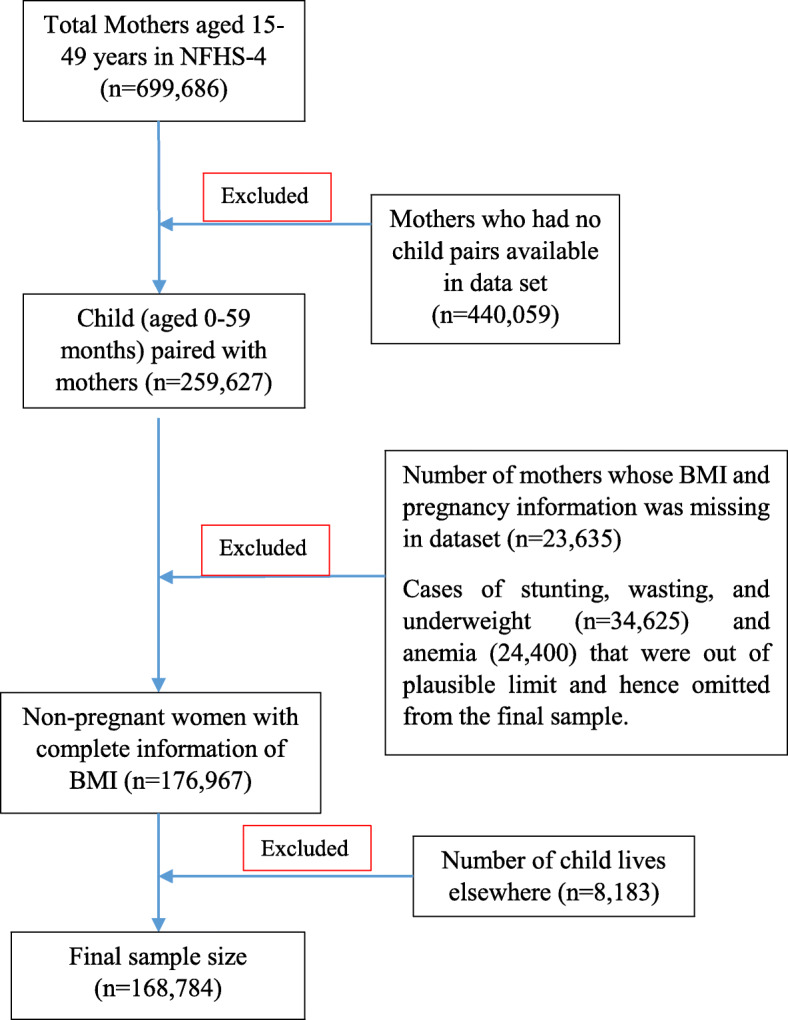
Flow chart for sample size selection

The independent variables included in this study were maternal, child, and household level factors. Mother’s factors included age (15–24, 25–34 and 35 years or more), age at first birth (less than 19, 20–29 and 30 years or more), educational level (no education, primary, secondary and higher), information on breastfeeding and cesarean section. Child level factors included child’s age (≤12, 13–23, 24–35, 36–47, and 48–59 months), sex of the child (male and female), received vitamin A and birth size of the child (average, smaller than average and larger than average). At last, household factors were categorized into the following parts; wealth index (poor, middle, and rich), Caste (Scheduled Caste, Scheduled Tribe, Other Backward Class and others), religion (Hindu, Muslim, and others), place of residence (rural and urban) and geographical regions (North, Central, East, Northeast, West, and South) of India. Figure 1 is showing the flow chart for sample size selection.

### Statistical analysis

To identify the mother and child pairs in the same household, women and child file data sets were merged for the analysis. Bivariate and binary logistic regression analyses were applied to assess the factors associated with the triple burden of malnutrition [20]. In bivariate analysis, a chi-square test was also performed to assess socio-demographic factors’ association with the triple burden of malnutrition at the household level. This study included only those variables in the multivariate analysis, which were statistically significant (*p* < 0.05) in bivariate analysis. The adjusted odds ratio with a 95% confidence interval was also presented in the results.

## Results

Table 1 represents the socio-demographic and other characteristics of the mother and children, 2015–16. It was found that 4.2%, 3.3%, 2.0%, and 7.8% of mother-child dyads were combinations of overweight and obese women with stunted (OM/SC), underweight (OM/UC), wasted (OMWC), and anemic children (OM/AC) respectively. Additionally, 5.7% of mother-child pairs were suffering from TBM, i.e., pairs were either (OM/SC) or (OM/UC) or (OM/WC) and (OM/AC). Nearly 3 in 10 mothers belong to the age group of 15–24 years. A total of 37% of mothers had age at first birth 19 years or less. Only about 1 in 10 mothers were having higher education. About 57% of mothers did not currently breastfeed, and 2 in 10 mothers went for a C-section method to deliver babies. Almost 22% of children were from the age group 48–59 months, and 14% were from the age group for 12 months or less. Nearly 47% of children were female, and 53% of them were male. Nearly 30% of the children did not receive vitamin A. Nearly 13% of the children were born smaller than the average size at birth. Nearly one-third of the households belonged to the rich wealth quintile.

**Table 1:** Socio-demographic profile of mother-child pairs in India, 2015–16

Variables	Frequency (%)
**OM/SC**	
Not stunted	162,286 (95.8)
Stunted	6498 (4.2)
**OM/UC**	
Not underweight	163,988 (96.7)
Underweight	4796 (3.3)
**OM/WC**	
Not wasted	165,927 (98.0)
Wasted	2857 (2.0)
**OM/AC**	
Not anemic	156,702 (92.2)
Anemic	12,082 (7.8)
**TBM**	
Normal	160,198 (94.3)
OM/SC/WC/UC & AC	8586 (5.7)
**Mother level factors**	
**Age of the women (in years)**	
15–24	51,072 (32.6)
25–34	101,665 (59.4)
> =35	16,047 (8.1)
**Age at first birth (in years)**	
< =19	58,881 (36.7)
20–29	104,788 (61.0)
> =30	5115 (2.4)
**Educational level**	
No education	49,762 (28.3)
Primary	24,297 (13.9)
Secondary	78,192 (46.9)
Higher	16,533 (10.9)
**Currently breastfeeding**	
No	94,147 (56.9)
Yes	74,637 (43.1)
**C-section delivery**	
No	144,101 (82.0)
Yes	24,683 (18.0)
**Child level factors**	
**Child’s age (in months)**	
< =12	22,801 (13.5)
13–23	33,654 (19.9)
24–35	35,965 (21.4)
36–47	38,725 (23.0)
48–59	37,639 (22.3)
**Sex of the child**	
Male	89,311 (53.0)
Female	79,473 (47.0)
**Received Vitamin A**	
No	54,891 (29.6)
Yes	113,893 (70.4)
**Size at birth**	
Average	117,136 (67.9)
Larger than average	28,890 (19.3)
Smaller than average	22,758 (12.8)
**Household level factors**	
**Wealth index**	
Poor	80,427 (45.2)
Middle	34,276 (20.1)
Rich	54,081 (34.7)
**Caste**	
Scheduled Caste	32,101 (21.7)
Scheduled Tribe	31,922 (10.1)
Other Backward Class	66,957 (44.1)
Others	37,804 (24.1)
**Religion**	
Hindu	124,060 (79.4)
Muslim	25,541 (15.8)
Others	19,183 (4.9)
**Place of residence**	
Urban	42,041 (29.1)
Rural	126,743 (70.9)
**Region**	
North	32,733 (13.4)
Central	48,169 (26.3)
East	35,225 (25.6)
Northeast	23,235 (3.5)
West	12,001 (12.8)
South	17,421 (18.4)

*OM/SC* Overweight/obese mother and stunted child, *OM/WC* overweight/obese mother and wasted child, *OM/UC* overweight/obese mother and underweight child, *OM/AC* overweight/obese mother and anemic child and Trible burden of Malnutrition (OM/SC/WC/UC &AC), *TBM* Triple Burden of Malnutrition, *C-section* Caesarean section

**Fig. 2:**
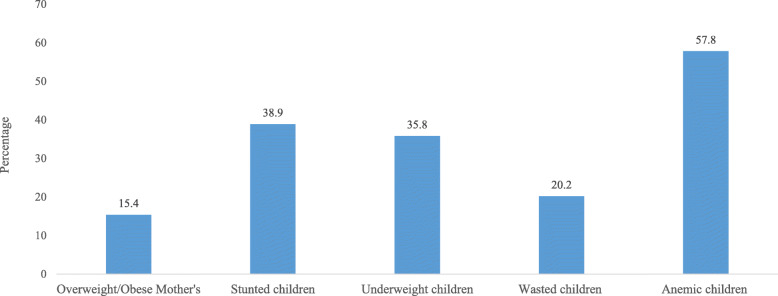
Percentage of nutritional status of the mothers and children in India, 2015–16

Figure 2 represents the percentage of the nutritional status of mothers and children in India. It was found that about 15.4% of women were overweight/obese. Nearly 38.9, 35.8, and 20.2% of children were stunted, underweight, and wasted, respectively. Almost 57.8% of children were anemic in India.

Table 2 presents results for the bivariate associations of background characteristics with TBM among mother-child pairs in India. TBM was highest among women who were more than 35 years (8.5%) of age than women in any other age group. Bivariate association indicates that TBM increases with age at first birth of mother, education level of mothers, child’s age, and wealth index. Among mothers with higher education levels, 8% of TBM was recorded. Current breastfeeding was another predictor of TBM; mothers who were currently breastfeeding had a lower prevalence of TBM. Cesarean-section (C-section) delivery is another predictor of TBM. The prevalence of TBM is nearly double when mothers had C-section delivery (9.8%) than when mothers had normal delivery (4.8%). TBM is lowest (4.2%) when the child’s age is equal to or less than a year than a child of any age group. Male children (5.8%) were having a higher burden of TBM than female children. Children whose birth size was larger than average were having a higher prevalence of TBM (6%) than children with any other birth size. Mother-child pairs which belong to the rich wealth quintile had the highest percentage of TBM (8.5%) than mother-child pairs of any other wealth quintile category. Mother-child pairs from another caste category (6.8%) and mother-child pairs that belong to the Muslim religion (7.8%) had a higher percentage of TBM than any other caste and religion category. Mother-child pairs from the urban place of residence had a higher prevalence of TBM (9%) than mother-child pairs from rural areas (4.3%). Mother-child pairs that belong to the southern region (8.6%) had the highest prevalence of TBM than mother-child pairs residing in any other region.

**Table 2:** Bivariate associations of background characteristics with triple burden of malnutrition among mother-child pairs in India

Variables	Triple Burden of Malnutrition (%)	Chi-square value (***p***-value)
**Mother level factors**		
**Age of the mother (in years)**		
15–24	3.87	278.48 (0.0001)
25–34	6.26	
> =35	8.50	
**Age at first birth (in years)**		
< =19	4.71	101.82 (0.0001)
20–29	6.10	
> =30	9.26	
**Educational level**		
No education	3.88	150.17 (0.0001)
Primary	4.91	
Secondary	6.43	
Higher	7.97	
**Currently breastfeeding**		
No	6.15	12.90 (0.0001)
Yes	5.02	
**C-section delivery**		
No	4.76	616.19 (0.0001)
Yes	9.79	
**Child level factors**		
**Child’s age (in months)**		
< =12	4.23	54.72 (0.0001)
13–23	5.14	
24–35	5.60	
36–47	6.68	
48–59	6.01	
**Sex of the child**		
Male	5.81	9.70 (0.0020)
Female	5.50	
**Received Vitamin A**		
No	4.93	35.60 (0.0001)
Yes	5.98	
**Birth size**		
Average	5.57	7.23 (0.0270)
Larger than average	5.96	
Smaller than average	5.72	
**Household level factors**		
**Wealth index**		
Poor	3.04	1000 (0.0001)
Middle	6.60	
Rich	8.55	
**Caste**		
Scheduled Caste	5.34	292.21 (0.0001)
Scheduled Tribe	2.88	
Other Backward Class	5.83	
Others	6.82	
**Religion**		
Hindu	5.17	329.61 (0.0001)
Muslim	7.80	
Others	6.82	
**Place of residence**		
Urban	9.01	803.37 (0.0001)
Rural	4.30	
**Region**		
North	5.83	687.55 (0.0001)
Central	5.02	
East	3.63	
Northeast	3.29	
West	7.33	
South	8.59	

*C-section delivery* Cesarean section delivery

Table 3 presents the result of multivariate logistic regression. Mothers whose age was 35 years and above were having a significantly higher likelihood of experiencing TBM than mothers from age group 15–24 years [AOR: 2.56, *p* < 0.01]. Mothers whose age at first birth was 30 years and above were 14% significantly less likely to suffer from TBM than mothers whose age at first birth was 19 years or less [AOR: 0.86, *p <* 0.01]. Mothers with educational status up to secondary level were 15% [AOR: 1.15, *p <* 0.01] more likely to suffer from TBM than mothers who had no education. Mothers who delivered through C-section had higher odds for suffering from TBM than mothers who had normal delivery [AOR: 1.57, *p <* 0.01]. Children aged 36–47 months had 67% more likely to suffer from TBM than children aged 1 year or less. Sex of the child and Vitamin A status did not have any significant association with TBM. Children whose birth size was smaller than average were 12% more likely to suffer from TBM than children with average birth size [AOR: 1.12, *p* < 0.01]. Mother-child pairs from the rich wealth quintile were 93% more likely to suffer from TBM than mother-child pairs from the poor wealth quintile [AOR: 1.93, *p* < 0.01]. Mother-child pairs from Scheduled Tribe were 25% less likely to suffer from TBM than mother-child pairs from other castes. Mother-child pairs from the Muslim religion were 58% more likely to suffer from TBM than mother-child pairs from the Hindu religion [AOR: 1.58, *p* < 0.01]. Women-child pairs from rural areas were 28% less likely to suffer from TBM than mother-child pairs from urban areas [AOR: 0.72, *p <* 0.01]. Mother-child pairs from the southern region were 41% more likely to suffer from TBM than mother-child pairs from the northern region [AOR: 1.41, *p <* 0.01].

**Table 3:** Odds ratio for triple burden of malnutrition by background characteristic for mother-child pairs in India, 2015–16

Variables	AOR (95% CI)
Mother level factors	
**Age of the mother (in years)**	
15–24®	
25–34	1.65***(1.56–1.76)
> =35	2.56***(2.34–2.80)
**Age at first birth (in years)**	
< =19®	
20–29	0.90***(0.85–0.95)
> =30	0.86**(0.76–0.97)
**Educational level**	
No education®	
Primary	1.12***(1.04–1.22)
Secondary	1.15***(1.08–1.23)
Higher	1.04 (0.95–1.14)
**Currently breastfeeding**	
No	0.90***(0.84–0.95)
Yes®	
**C-section delivery**	
No®	
Yes	1.57***(1.49–1.66)
**Child level factors**	
**Child’s age (in months)**	
< =12®	
13–23	1.24***(1.13–1.36)
24–35	1.40***(1.28–1.54)
36–47	1.67***(1.52–1.84)
48–59	1.54***(1.39–1.70)
**Sex of the child**	
Male®	
Female	0.97 (0.93–1.01)
**Received Vitamin A**	
No®	
Yes	1.02 (0.97–1.07)
**Birth size**	
Average®	
Larger than average	1.00 (0.95–1.06)
Smaller than average	1.12***(1.04–1.19)
**Household level factors**	
**Wealth index**	
Poor®	
Middle	1.79***(1.68–1.92)
Rich	1.93***(1.8–2.07)
**Caste**	
Scheduled Caste	1.11***(1.04–1.20)
Scheduled Tribe	0.75***(0.68–0.82)
Other Backward Class	1.04 (0.98–1.10)
Others®	
**Religion**	
Hindu®	
Muslim	1.58***(1.49–1.67)
Others	1.34***(1.24–1.46)
**Place of residence**	
Urban®	
Rural	0.72***(0.68–0.76)
**Region**	
North®	
Central	1.07**(1.04–1.15)
East	0.83***(0.76–0.90)
Northeast	0.86***(0.78–0.94)
West	1.20***(1.10–1.31)
South	1.41***(1.31–1.53)

*®* References, *AOR* Adjusted Odds Ratio, *CI* confidence Interval

***if *p* < 0.01, **if *p* < 0.05, *if *p* < 0.1

*C-section delivery* Cesarean section delivery

**Fig. 3:**
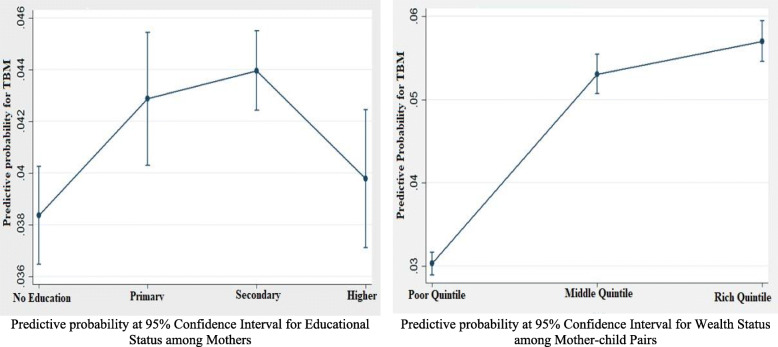
Predictive probability for selected background characteristics for TBM among Mother-child pairs in India

Figure 3 represents the predictive probability for selected socioeconomic covariates for TBM among mother-child pairs in India. The estimates were presented after controlling all the other background variables at their mean values (the estimates are obtained after the post-estimation command). It was found that mothers with secondary education level were having a higher probability of suffering from TBM, and interestingly the probability shattered down for mothers having a higher educational level. Additionally, mother-child pairs from rich wealth status had a higher probability of suffering from TBM.

## Discussion

This study attempted to explore the coexistence of TBM among mother-child pairs in India. Overall, the TBM prevalence was 5.7% among mother-child pairs, which was lower than the 7% prevalence of TBM in Nepal [20]. The burden of malnutrition at the household level among mother-child pairs was 4.2% compared to pairing overweight or obese mothers with the stunted child only, which is higher than Nepal and Pakistan, lower than Myanmar, and equal to Bangladesh [25]. Another study found that the double burden of overweight/obese mothers and stunted children was 11 and 4% in rural Indonesia and rural Bangladesh, respectively [26]. Further, this study found that the burden of overweight/obese mothers and underweight children was 3.3%, overweight/obese mothers and wasted children were 2%, and overweight/obese mothers and anemic children were 7.8% in the same household.

Numerous studies have highlighted the risk factors for the double burden of malnutrition [25–28]; however, minimal literature is available in the public domain for TBM among mother-child pairs [20].

The study found that various maternal, child, and household factors were associated with TBM among mother-child pairs in India. The rising age of the mother is one of the prominent risk factors for TBM. Various studies have noted a positive association between increasing women’s age and the rise in obesity [29]. The high prevalence of obesity among mothers in the higher age group is the plausible reason for higher TBM among mothers of higher age. During later age, physical activity among women declines, along with the metabolic rate, leading to an increase in obesity. Furthermore, the energy requirement decreases; therefore, even regular or routine eating may lead to weight gain among women at later ages [30]. Moreover, newly-married women at a young age are more health-conscious and involved in more physical activity than women at older ages with children, which may further be attributed to obesity among women at later ages [31]. Increasing age in mothers is correlated with higher parity, which is another contributory factor for overweight and obesity [32]. Women gain weight during pregnancy, and weight loss does not occur in the post-partum period, which is another cause of obesity among women in a higher age group [33]. Another important finding relates TBM to the age of the mother at first birth. As age at first birth increases, the TBM among mother-child pairs decreases. Previous studies noticed an association between increasing the mother’s age at first birth and improving child undernutrition [34]. Child development has been linked to the mother’s age in various settings [35]. Studies have found that an increase in a mother’s age is associated with an increase in women’s autonomy, which positively affects child growth [36–38]. However, a study found that decision-making among women does not improve stunting or wasting among children [39]. Another study revealed that women with low acceptance in couple relations were less likely to have stunted or wasted children [40].

Mother’s education is another prominent covariate that has an association with TBM. Results concluded that primary and secondary level educated mothers were more likely to have a higher risk for TBM than uneducated mothers. However, anemia among children decreases with an increase in education among mothers [18], it is increasing in obesity prevalence with an increase in mother’s education [41] that is contributing to the higher levels of TBM among mother-child pairs [20]. The prevalence of obesity was higher among educated women than in uneducated women [42], contributing to increased TBM among highly educated women. Women with high education levels generally have higher wealth status than less-educated women [43], and higher wealth in a household is positively linked with higher odds of obesity among women [4]. C-section delivery is another contributory factor for TBM among mother-child pairs. Results showed that mothers who delivered a baby through C-section were more likely to suffer from TBM. It is widely studied that children born through C-section deliveries are less likely to be breastfed [44–46] and suffer from undernutrition [47]. It is further noted that mothers with C-section have difficulties with breastfeeding (Hobbs et al., 2016). Moreover, previous studies have commented that children who got delivered via C-section face early breastfeeding cessation [45, 46]. Furthermore, it is explored through previous studies that late initiation of breastfeeding due to C-section promotes poor child growth [45]. Few other studies have associated C-section deliveries with poor developmental growth outcomes among children [27, 47]. Moreover, C-section is also attributed to the increased risk of obesity among women [48]. All the above factors lead to higher TBM among mother-child pairs when the mother goes through C-section delivery.

Child’s age and birth size are the two child-related factors that were found to be significantly associated with the risk of TBM. Higher child age is associated with a high risk of TBM. Malnutrition among children increases with age [31] and can be attributed to higher TBM among them. Previous studies also highlighted that growth faltering among children is higher later [17]. A higher prevalence of stunting, wasting, and underweight at later ages is the plausible cause of the high risk of TBM among children at later ages. Not only undernutrition, but anemia is also higher among children in the high age group than children with lower age [49], thus confirming the high risk of TBM among children with a high age group. It is clearly understood that children aged above 1 year are at high risk of TBM than children below 1 year. As a child continues to grow after birth, the body grows rapidly and requires nutritious food that may not be fulfilled by regular diet, and hence children are more anemic, stunted, and underweight as they grow [49]. Smaller than average birth size is positively associated with the risk of TBM. Previous studies have attributed the low birth weight to stunting, wasting, and underweight [50, 51]. A study found that children with low birth weight were at 20% higher risk of being stunted [52]. Low size at birth is associated with a smaller amount of fundamental development supplements; nutrients A, zinc, and iron [53]. Thus, low birth weight babies were more malnourished as compared to high birth weight babies.

This study found that the TBM is higher among wealthier households than in poor households. As TBM was measured by creating mother-child pairs, it is crucial to understand what contributes to higher TBM in more affluent households; is it undernutrition and anemia among children or obesity among mothers? Studies have concluded that obesity is higher among richer women than in poor women [54, 55], and undernutrition and anemia among children are higher among poor children than in richer children [56]. Therefore, it is understood from the available literature that a higher prevalence of obesity among women in richer households is contributing to the TBM among mother-child pairs. It is a well-established notion that people in wealthier households tend to follow a sedentary lifestyle and are engaged in less labour intensive work, and consume more energy due to greater purchasing power; all of these lead to higher rates of obesity among them [57]. Contrary, it was also confirmed that low socioeconomic status might be related to limited food intake and combined with high manual labor, leading to a negative energy intake contributing to low TBM among women from poor households [58]. Consuming high energy-dense foods among mother-child pairs from wealthier households may be another reason for higher TBM among them than mother-child pairs from poor households, as previous studies have indicated that consuming high energy-dense food may well be attributed to obesity among women [59]. Results elaborated that TBM was lower among mother-child pairs belonging to Scheduled Tribes. Previous studies are also in line with noticing a higher prevalence of underweight and lower prevalence of overweight/obesity among the ST groups [59]. This may be explained by the situation of the tribe population, being primarily exposed to discrimination and a socioeconomically disadvantaged group [60].

The study found that TBM is higher among mother-child pairs in urban areas than in rural areas. Urban women have higher levels of obesity than their rural counterparts [29]; thus, TBM is higher among mother-child pairs in urban areas than in rural areas. Urbanization impacts the way of life, thus increasing the obesity levels among women in urban areas [61]. Sedentary behaviors and inadequate physical activity have been documented as risk factors for overweight/obesity among women residing in urban areas [62]. Moreover; western culture has long lasted impacts on obesity in urban India because of the opening of several fast-food chains and stalls across the cities [29]. The result noted a higher prevalence of TBM in Southern and Western regions than in India’s Northern region. It is evident from previous studies that education and wealth status in the Southern and Western regions are higher than in the Northern region (Chandra, 2019). Higher wealth and education, as discussed above, can explain the high risk of TBM among mother-child pairs in Southern and Western regions.

The study has several strengths. The anthropometric measures such as height and weight used to calculate BMI for mothers were measured with standard procedures by a team of highly trained investigators. Since the survey has a large sample size, the findings can be generalized to all households in India. As studies have highlighted the issue of post-pregnancy weight gain among mothers [63], this study dropped those mothers from the sample who delivered a baby in the last 2 months. Moreover, this study also dropped pregnant mothers’ cases with previous birth histories as weight gain during pregnancy is a well-researched phenomenon [64]. Despite several strengths, this study has some noteworthy limitations. Firstly, this study could not establish a plausible causal pathway of the association between explanatory and dependent variables and add only to the studies related to TBM’s prevalence and factors. Furthermore, the nutritional status of the mother was assessed using BMI only. Methods such as waist-hip ratio, bioelectrical impedance technique, DESA, and skinfold thickness are more accurate than BMI to assess overweight/obesity [20].

## Conclusion

The study is important in highlighting the risk factors for TBM among mother-child pairs in India. This study highlights the issue of TBM by measuring undernourishment (stunting, wasting, and underweight) and anemia among children and overweight/obesity among women in the same households. At first, further research is needed to identify the causes and associated risk factors of TBM. On one side, a household is suffering from undernutrition and anemia among children, while on the other side, the same household is also suffering from overweight/obesity among mothers. The study found that the TBM exists among mother-child pairs in India. Various factors were associated with TBM among mother-child pairs, namely: age of the mother, age at first birth, education level of the mother, Child age, wealth index, place of residence, and regions of residence. There is a need for policies and scaled-up programs to provide nutritional education so that people start understanding the value of nutritious food and start investing in nutritious food choices. Food security and nutrition policies and programs must consider the specific needs and priorities of mother and children separately and target interventions in a gender-responsive way that leaves no one behind. Nutrition programs shall target undernutrition and anemia among children and obesity among women in the same household. It is important to promote public health programs that aim to create awareness about the harmful effects of sedentary lifestyles. Isolated focus on either undernourished children or overweight/obese women may not be adequate, and it is suggested to tackle different nutritional problems at the same time. There is a pressing need to implement nation-wide maternal health promotion interventions and nutrition education programs, which would be a good strategy to prevent overweight/obesity among women and undernutrition among children under 5 years of age in India. Promoting mutually inclusive nutrition interventions shall be the priority as TBM is higher among the wealthier families.

## Data Availability

The study utilizes secondary sources of data that are freely available in the public domain through https://dhsprogram.com/methodology/survey/survey-display-355.cfm. Those who wish to access the data may register at the above link and thereafter can download the required data free of cost.

## Abbreviations

TBM: Triple Burden of Malnutrition
NFHS: National Family Health Survey
FAO: Food and Agriculture Organization
UNICEF: United Nations International Children’s Emergency Fund
MoHFW: Ministry of Health and Family Welfare
PSU: Primary Sampling Unit
CEB: Census Enumeration Block
PPS: Probability Proportional to Size
IIPS: International Institute for Population Sciences
HAZ: Height-for-Age
WHZ: Weight-for-Height
WAZ: Weight-for-Age
BMI: Body Mass Index
WHO: World Health Organization
OM: Overweight/Obese Mother
SC/UC/WC/AC: Stunted Child/Underweight Child/ Wasted Child/ Anemic Child
C-Section: Cesarean Section
AOR: Adjusted Odds Ratio
